# Netrank: network-based approach for biomarker discovery

**DOI:** 10.1186/s12859-023-05418-6

**Published:** 2023-07-29

**Authors:** Ali Al-Fatlawi, Eka Rusadze, Alexander Shmelkin, Negin Malekian, Cigdem Ozen, Christian Pilarsky, Michael Schroeder

**Affiliations:** 1 Biotechnology Center (BIOTEC), Center for Molecular and Cellular Bioengineering, Technische Universität Dresden, Dresden, Germany; 2 Center for Scalable Data Analytics and Artificial Intelligence (ScaDS.AI), TU Dresden, Dresden, Germany; 3 University of Kufa, Najaf, Iraq; 4 Department of Surgical Research, Universitätsklinikum Erlangen, Erlangen, Germany

**Keywords:** Biomarker, Cancer, Protein networks, RNA, Gene expression, R package

## Abstract

**Background:**

Integrating multi-omics data is fast becoming a powerful approach for predicting disease progression and treatment outcomes. In light of that, we introduce a modified version of the NetRank algorithm, a network-based algorithm for biomarker discovery that incorporates the protein associations, co-expressions, and functions with its phenotypic association to differentiate different types of cancer. NetRank is introduced here as a robust feature selection method for biomarker selection in cancer prediction. We assess the robustness and suitability of the RNA gene expression data through scanning genomic data for 19 cancer types with more than 3000 patients from The Cancer Genome Atlas (TCGA).

**Results:**

The results of evaluating different cancer type profiles from the TCGA data demonstrate the strength of our approach to identifying interpretable biomarker signatures for cancer outcome prediction. NetRank’s biomarkers segregate most cancer types with an area under the curve (AUC) above 90% using compact signatures.

**Conclusion:**

In this paper we provide a fast and efficient implementation of NetRank, with a case study from The Cancer Genome Atlas, to assess the performance. We incorporated complete functionality for pre and post-processing for RNA-seq gene expression data with functions for building protein-protein interaction networks. The source code of NetRank is freely available (at github.com/Alfatlawi/Omics-NetRank) with an installable R library. We also deliver a comprehensive practical user manual with examples and data attached to this paper.

## Background

In the field of oncology, gene expression serves as a potent indicator of disease progression and its outcome prediction. Over decades, microarrays and RNA sequencing have been used extensively to investigate profiles in the form of biomarker signatures by quantifying the expression level of genes in the RNA data in cancer patients compared to the average in healthy individuals. To this end, several classical statistical methods have been introduced, such as DESeq2 [1], edgeR [2], and limma [3]. We urge that a trustworthy biomarker signatures should be interpretable, compact, robust to data changes, and neither overfitted nor biased toward the original data. However, due to the complexity of diseases and the high dimensionality of the data analyzed, difficulties arise when attempting to deliver a causal and interpretable model for predicting disease outcomes and progression. Perhaps the main limitation of the classical methods is that they evaluate biomarkers independently, regardless of their functional and statistical dependencies. This necessitates using complementary techniques to address and handle these difficulties, such as network analysis.

Network science has provided a useful account of exploring other aspects in assessing the biomarker significance besides the statistical correlation with a phenotype, such as molecular and functional interactions. Previously, we developed a random surfer model to integrate protein interactions with expression and phenotypic information to rank biomarkers according to their effectiveness in predicting cancer progression [4, 5]. Inspired by the concept of ten cancer hallmarks [6], our objective was to explore the possibility of defining a universal cancer biomarker signature by focusing on the common features between cancer types and disregarding the differences [4]. This effort resulted in finding a biomarker signature of 50 genes that were interpretable and robust in predicting cancer outcomes in general, regardless of the type, with an area under the curve between 80% and 90% for different datasets [4]. However, this signature cannot be extrapolated to distinguish between different cancer phenotypes or profiles, as it focuses only on the common mechanisms and functions.

**Fig. 1:**
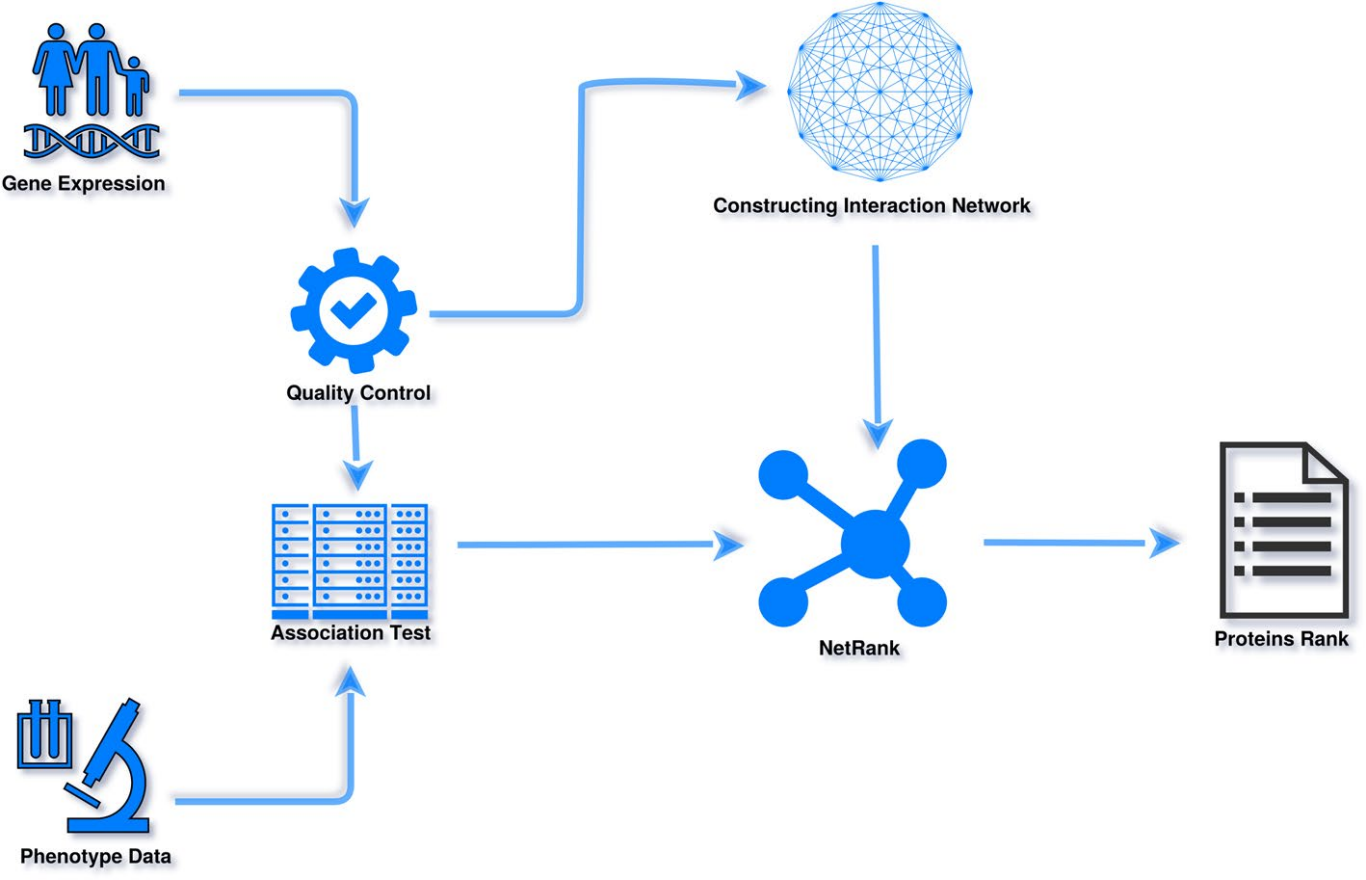
A flowchart to explain the primary processes in the implemented pipeline

Our work aims to extend the implementation and application of NetRank to differentiate different cancer phenotype and deliver an open source R implementation for the algorithm with complete functionality, including pre-and post-processing of RNA-seq gene expression data, see Fig. 1. We evaluate our algorithm and implementation by differentiating between 19 cancer types in 3388 patients (data obtained from the Cancer Genome Atlas (TCGA) https://portal.gdc.cancer.gov/).

## Implementation

### NetRank

**Fig. 2:**
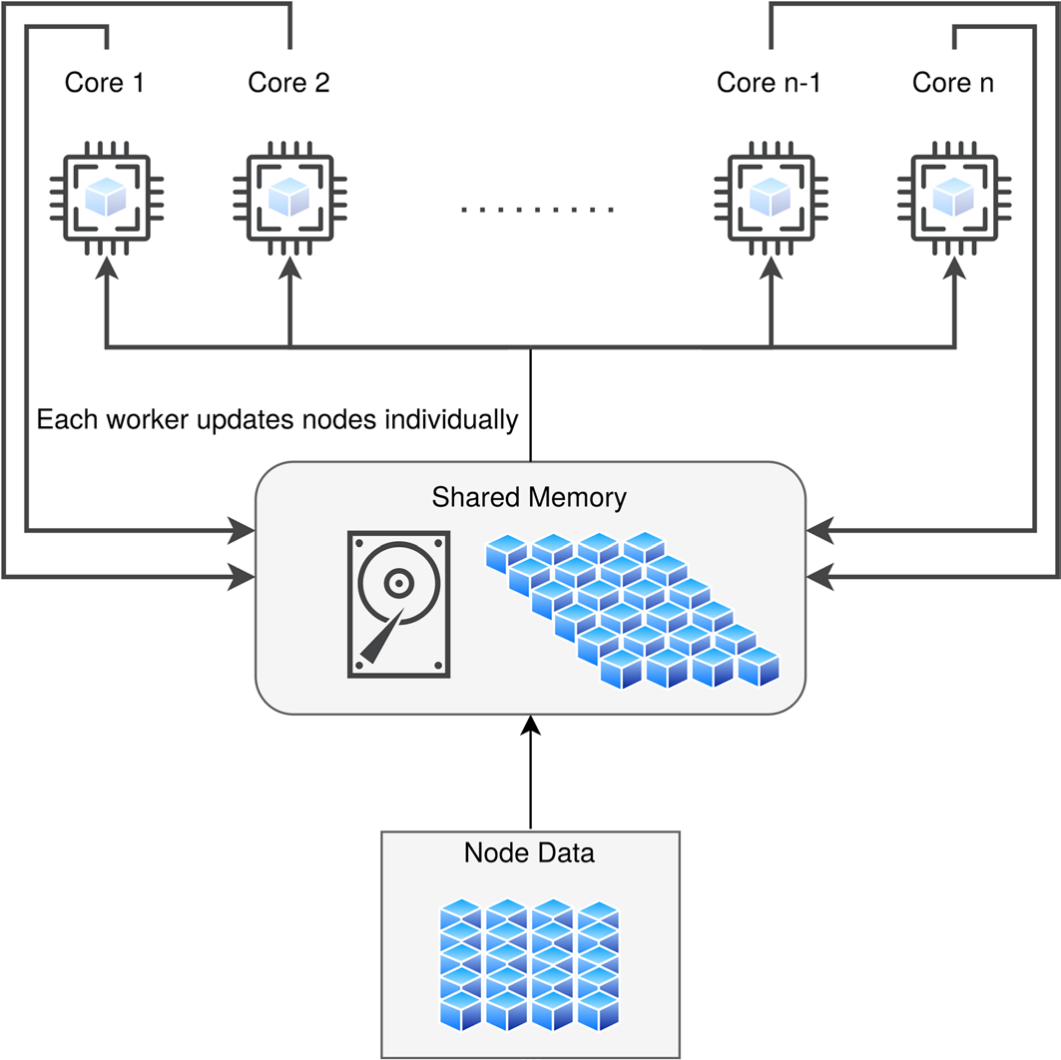
The parallel processing mechanism for the implemented pipeline

NetRank is a random surfer model for biomarker ranking inspired by Google’s PageRank algorithm. NetRank integrates protein connectivity (e.g., co-expression, signaling pathways, biological functions, co-localization, fusion, co-occurrence) with their statistical phenotypic correlation, see Eq. (1). It favors proteins strongly associated with the phenotype and connected to other significant proteins. The algorithm is implemented with the R version 3.6.3. The provided implementation allows parallel processing with any number of cores by utilizing shared memory using the packages “bigstatsr”, “foreach” and “doparallel”, see Fig. 2.

1 $$\begin{aligned} r_j^n= (1-d)s_j+d \sum _{i=1}^N \frac{m_{ij}r_i^{n-1}}{degree^i} \text {,} \quad 1 \le j \le N \end{aligned}$$

**r:** ranking score of the node (gene). **n:** number of iterations. **j:** index of the current node. **d:** damping factor (ranging between 0 and 1); defines the importance (weights) of connectivity and statistical association. **s:** Pearson correlation coefficient of the gene. **degree:** the sum of the output connectivities for the connected nodes. **N:** number of all nodes (genes). **m:** connectivity of the connected nodes.

### Interaction networks

We implemented and evaluated NetRank to work with two types of networks: biological precomputed networks (protein-protein interaction) and computationally computed networks (co-expression of genes). For the first one, we used the database STRINGdb, which covers predicted and known biological interactions between proteins through the R package “STRING v10” [7]. For the latter, we implemented a workflow to construct a co-expression network using the weighted gene correlation network analysis (WGCNA) method [8] through the R package “WGCNA” version 1.71 [9].

### Dataset

**Fig. 3:**
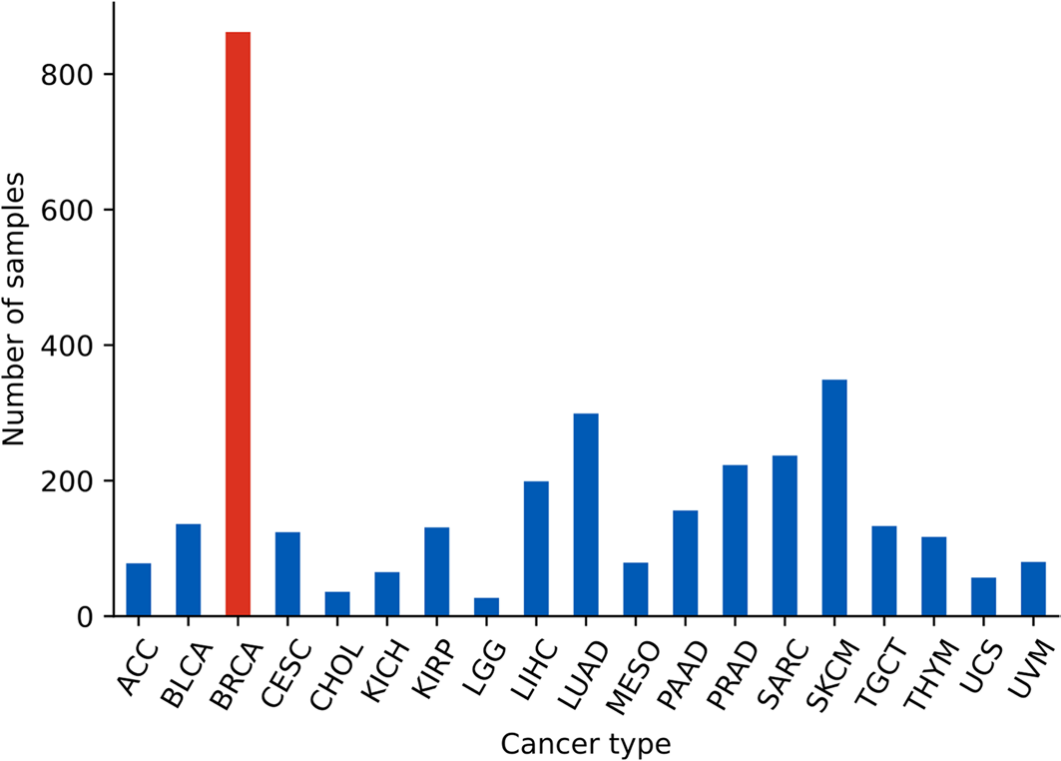
Overview of the final dataset with 3388 samples covering 19 cancer types. Breast cancer (BRCA, in red color) has more data with 862 samples, while lower grade glioma (LGG) has the smallest size with 27 samples. For the complete list of abbreviations, see Table 3

We obtained RNA gene expression data on 05/08/2022 from the Cancer Genome Atlas (TCGA), which initially consisted of 20,531 genes and 11,069 samples. We kept only 8603 samples after removing those who were duplicated or had missing values in the expression levels. Of these, we used only 3388 samples that were manually reviewed and approved in TCGA clinical follow-up. These 3388 were covering 19 cancer types, see Fig. 3. We normalized the expression data using the MinMaxScaler function of scikit-learn package version 1.0.2 [10]. We randomly split the data for each cancer type into a development set (70%) and a test set (30%). Using the development set, the interaction networks are constructed as described previously, and the Pearson correlation with the phenotypes is determined by the correlation function of the WGCNA package version 1.71. Then to the test set was utilized for evaluation using the principal component analysis (PCA) and the support vector machine (SVM) on each cancer type.

## Results and discussions

A case study of 19 cancers with 3388 individuals was conducted to evaluate the performance of the NetRank algorithm as a feature selection and ranking method for RNA expression data. In our primary analysis, we visualized breast cancer results because it contains the highest number of samples. In this, 862 (breast cancer samples) were labeled as cases and 2526 (other cancer types) as controls. Out of these, 618 (cases) and 1753 (controls) were used in the development set for the feature selection and ranking, while 244 (cases) and 773 (controls) were kept unseen for evaluation purposes (test set). This corresponds to the usual data division of 70% and 30% between the development and test sets to avoid over-fitting.

Furthermore, the analysis was replicated to the other 18 cancer types with the same setup and configuration, and the final results are reported accordingly in this section.

### The results of the STRINGdb and the co-expression networks are correlated

**Fig. 4:**
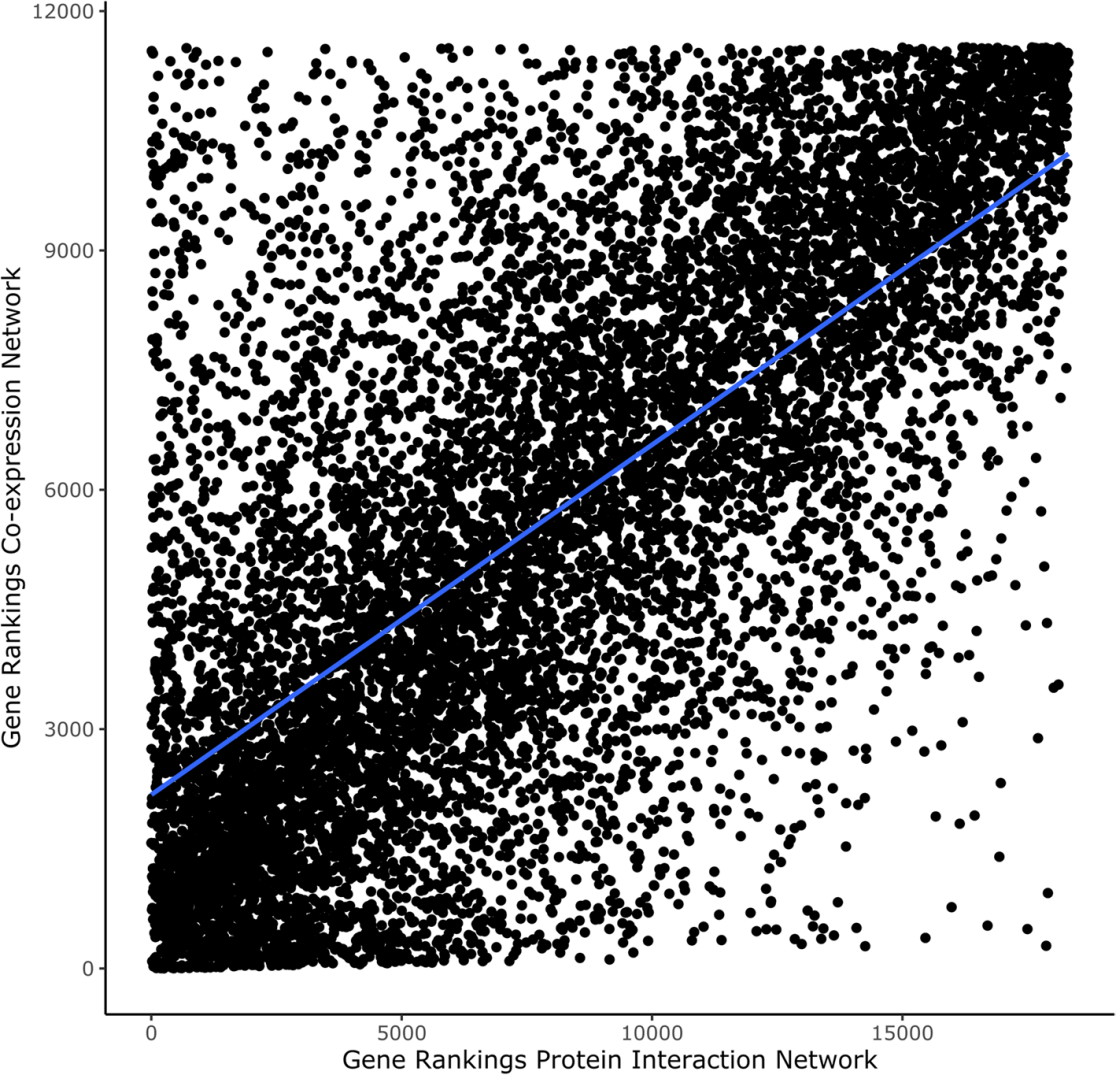
The correlation of the gene rankings generated by NetRank when applied to a protein-protein interaction network and a gene co-expression network. According to the figure, the rankings are significantly correlated, with a Pearson R-value of 0.676

We performed NetRank on two kinds of networks: the one obtained from StringDB and the co-expression network that is constructed using WGCNA (see the implementation section). Wecompared the results. STRINGdb was fetched directly, while WGCNA was locally computed using our curated dataset. For each network, we ranked proteins according to their suitability for breast cancer prediction. What stands out is the correlation in ranking proteins according to their suitability for breast cancer prediction between the STRINGdb and the WGCNA network (Pearson’s R-value = 0.68), see Fig. 4. Accordingly, we can infer that the results are comparable when using different types of networks.

### Proteins with high NetRank scores serve as informative biomarkers

NetRank scores the proteins’ potential in cancer type prediction, according to their connectivities and associations. For breast cancer data, using the development set, we picked the top 100 proteins with a P-value of association below 0.05 and have the highest NetRank score in separating breast cancer from the other cancer types. Then, we evaluated their performance using the test set by performing principal component analysis (PCA), a linear and simple dimensionality reduction method, and support vector machine (SVM), a nonlinear machine learning approach.

**Fig. 5:**
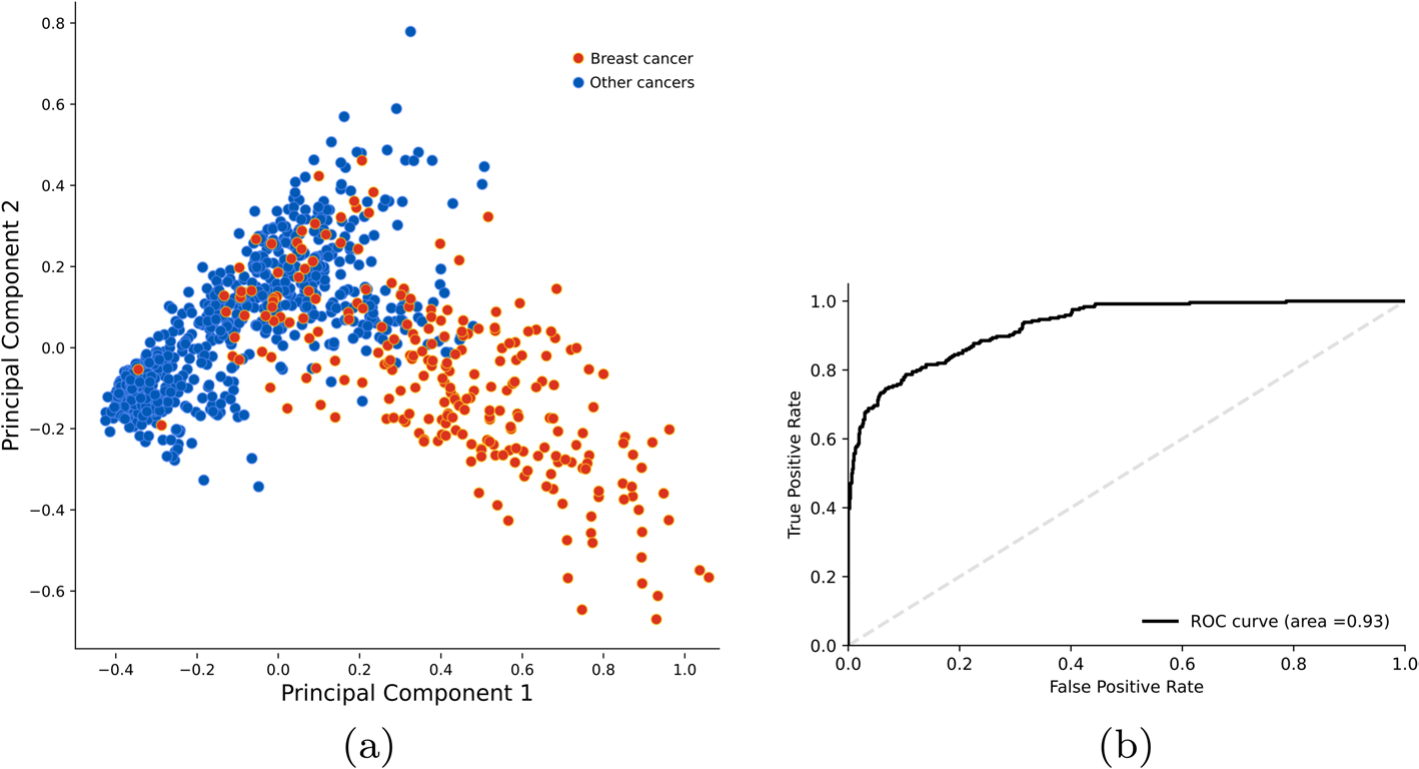
**a** PCA plot of the TCGA samples generated by using the top hundred proteins selected based on their NetRank scores. The red dots are breast cancer samples, while the blue refers to the other 18 cancer types in the dataset. The two different clusters can be visually separated very easily. **b** The ROC curve of the first principle component. The high diagnostic ability of these proteins is confirmed by the AUC value of 0.93

What is striking is the significant segregation of individuals with breast cancer from the from the ones with other cancer types in the test set when using PCA, with an area under the ROC curve (AUC) of 93% for the first principal component (see Fig. 5). Using the same features, SVM classifies the data nearly perfectly with accuracy and an F1 score of 98% (see Fig. 1).

The functional enrichment analysis on the breast cancer signatures shows that selected biomarkers harbor biological relevance through enhancing the protein-protein enrichment significance with 88 enriched terms in 9 relevant categories, compared with only nine terms if we choose the proteins according to their statistical associations only. The list of genes is provided in the Appendix.

### Other cancer types

In the same manner, we generated a feature set for each cancer type independently and evaluated them with machine learning using SVM. As is shown in Table 1, the method is nearly perfect for all cancer types in the Cancer Genome Atlas (TCGA) with an AUC and accuracy above 90% except for Cholangiocarcinoma (CHOL), Bladder Urothelial Carcinoma (BLCA) and Uterine Carcinosarcoma (UCS), which have a limited AUC of 82%, 79%, and 71%, respectively. All signatures are provided in the Appendix. Overall, our implementation introduces a powerful feature selection method with demonstrable results on cancer sequencing data for around 4000 patient.

**Fig. 6:**
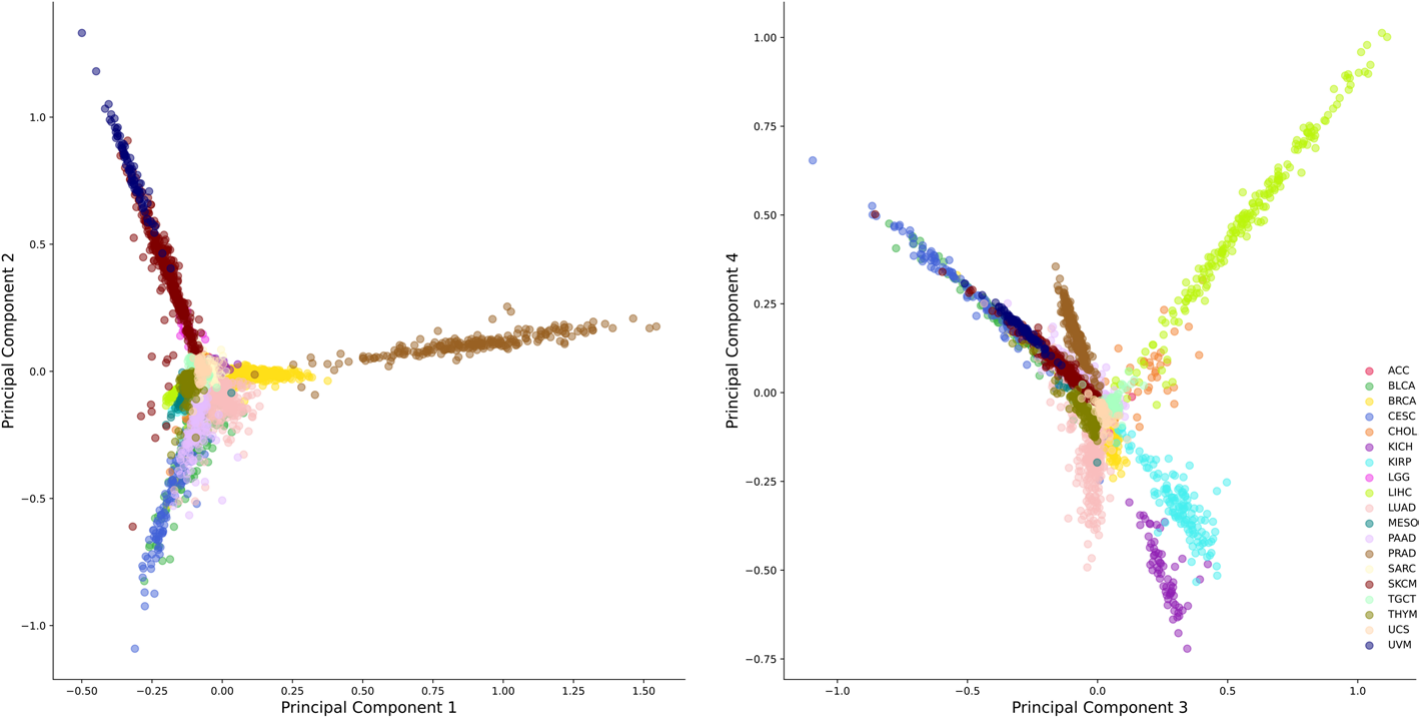
Principle component analysis on the expression data by using the best ten proteins from each analysis (19 cancer types) according to their NetRank scores. The two plots are for the same analysis but use different principle components on the x and y-axis. We visualize the first and second components on the left side, while on the right side, the third and fourth components. It is clearly visible that cancers are separated in these figures

Additionally, for visualization purposes, we picked the best ten biomarkers from each cancer type and ran the principal component analysis on them (171 unique proteins), using all data. As shown in Fig. 6, the selected biomarkers cluster the data and distinguish different cancer types very well.

**Table 1:** The performance of the SVMs that were trained with the top hundred genes as ranked by the Netrank on the development set and tested with the test set

Cancer type	Precision	Recall	F1_score	Accuracy	AUC	No. samples			
						Development set		Test set	
						Case	Control	Case	Control
ACC	0.99	0.95	0.97	0.99	0.95	57	2314	21	996
BLCA	0.97	0.79	0.85	0.98	0.79	95	2276	41	976
BRCA	0.97	0.97	0.97	0.98	0.97	618	1753	244	773
CESC	0.94	0.95	0.94	0.99	0.97	91	2280	33	984
CHOL	0.99	0.81	0.88	0.99	0.82	25	2346	11	1006
KICH	0.99	0.92	0.96	0.99	0.93	44	2327	21	996
KIRP	0.99	0.98	0.99	0.99	0.99	88	2283	43	974
LGG	1	0.95	0.97	0.99	0.95	16	2355	11	1006
LIHC	0.99	0.95	0.97	0.99	0.96	132	2239	67	950
LUAD	0.99	0.95	0.97	0.99	0.95	213	2158	86	931
MESO	0.97	0.89	0.93	0.99	0.9	54	2317	25	992
PAAD	0.92	0.95	0.93	0.99	0.96	120	2251	36	981
PRAD	0.99	0.99	0.99	0.99	0.99	141	2230	82	935
SARC	0.98	0.94	0.96	0.99	0.94	166	2205	71	946
SKCM	0.97	0.97	0.97	0.99	0.98	250	2121	99	918
TGCT	0.98	0.98	0.98	0.99	0.99	95	2276	38	979
THYM	0.99	0.97	0.98	0.99	0.97	83	2288	34	983
UCS	0.99	0.71	0.79	0.98	0.71	38	2333	19	998
UVM	0.97	0.99	0.98	0.99	1	61	2310	19	998

The numbers of positive and negative samples in the training and testing sets are reported

**Table 2:** Time taken, memory consumption, and CPU usage of the NetRank R package functions used on the development set of the breast cancer samples

Function name	Description	Time (min)	Memory (GB)	CPU usage
Fetch StringDB	Fetch data from STRINGdb	1.41	0.75	100% (1 core)
RunNetRank (StringDB)	Apply NetRank algorithm	19.02	0.53	1500% (15 cores)
Preprocessing	Preprocess expression data	29.9	0.53	100% (1 core)
BuildNetwork	Create the co-expression network	126	0.53	100% (1 core)
Postprocessing	Prepare the network for NetRank	6.3	0.05	100% (1 core)
RunNetRank (Co-expression)	Apply NetRank algorithm	8.7	0.08	1500% (15 cores)

**Table 3:** The abbreviations of cancer types that were used in Figs. 3, 6 and Table 1

Abbreviation	Cancer type
ACC	Adrenocortical carcinoma
BLCA	Bladder Urothelial Carcinoma
BRCA	Breast invasive carcinoma
CESC	Cervical squamous cell carcinoma and endocervical adenocarcinoma
CHOL	Cholangiocarcinoma
KICH	Kidney Chromophobe
KIRP	Kidney renal papillary cell carcinoma
LGG	Brain Lower Grade Glioma
LIHC	Liver hepatocellular carcinoma
LUAD	Lung adenocarcinoma
MESO	Mesothelioma
PAAD	Pancreatic adenocarcinoma
PRAD	Prostate adenocarcinoma
SARC	Sarcoma
SKCM	Skin Cutaneous Melanoma
TGCT	Testicular Germ Cell Tumors
THYM	Thymoma
UCS	Uterine Carcinosarcoma
UVM	Uveal Melanoma

### Performance evaluation

We measured the performance of the NetRank R package on the development set of breast cancer data, i.e. 618 case and 1753 control samples, using a computer with 15 cores. As can be seen in Table 2. This shows the effluence and speed of our implementation using normal computers.

## Conclusion

This application note has introduced an extended open-source implementation of the NetRank algorithm, a network-based approach for biomarker discovery in cancer prediction. By incorporating protein associations, co-expressions, and functions, along with phenotypic associations, NetRank has demonstrated its robustness and suitability for identifying interpretable biomarker signatures in various cancer types. Our approach is advantageous compared to correlation-based methods. It ensures that proposed biomarkers share a common function, avoiding unrelated and challenging interpretations.

This manuscript focuses on technical aspects and package usage, providing a case study demonstrating the network approach’s ability to identify meaningful outcomes. Emphasizing the functional relevance of biomarkers enhances understanding and opens avenues for targeted therapies or diagnostics. This paper contributes to the field by providing fast and efficient implementation of NetRank and comprehensive functionality for pre and post-processing RNA-seq gene expression data and building protein-protein interaction networks. The availability of the source code on GitHub and the accompanying R library allows for easy accessibility and further exploration by researchers.

## Data Availability

Code, user guide and data: github.com/Alfatlawi/Omics-NetRank. Project name: NetRank. Project home page: github.com/Alfatlawi/Omics-NetRank. Operating system(s): Platform independent. Programming language: R. Other requirements: R 3.6.3.

## Appendix

A file with a sheet for each cancer type containing the protein names that have the highest NetRank scores, which were used in the PCA and SVM in this paper.

## Abbreviations

AUC: Area under the curve
PCA: Principal component analysis
ROC: Receiver operating characteristic
SVM: Support vector machine
TCGA: The cancer genome atlas
